# Diagnostic Contexts of Echocardiographic Nonapical Window

**DOI:** 10.1016/j.jaccas.2024.102287

**Published:** 2024-03-11

**Authors:** Paolo Springhetti, Giovanni Benfari, Stefano Nistri, Elena Maria Santina Jannello, Giulia Elena Mandoli, Luigi Badano, Flavio Luciano Ribichini, Denisa Muraru

**Affiliations:** a Department of Medicine, Division of Cardiology, University of Verona, Verona, Italy; b Department of Cardiology, Istituto Auxologico Italiano, IRCCS, Milan, Italy; c Department of Cardiology, CMSR, Vicenza, Italy; d Division of Cardiology, University of Siena, Siena, Italy; e Department of Medicine and Surgery, University of Milano-Bicocca, Milan, Italy

**Keywords:** aortic stenosis, right parasternal approach, right parasternal view

## Abstract

The long-established utility of multiwindow interrogation in echocardiography (suprasternal notch, right and left sternal border, apex, and subxiphoid) is sometimes not systematically implemented in routine practice. This case series emphasizes the pivotal importance of such practice for the systematic assessment of aortic valve stenosis and in the evaluation of left ventricular outflow tract and the aorta.

## Graphical abstract

**Figure:**
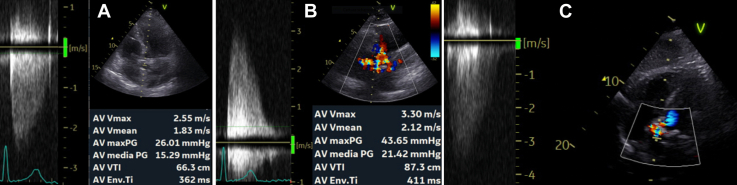

The cornerstone of the hemodynamic evaluation in patients with calcified or prosthetic aortic valve relies on the Doppler continuity equation, which is profoundly affected by the proper alignment of the ultrasound beam parallel with the transvalvular blood flow jet. Improper alignment could result in underestimation of the peak jet velocity and mean gradient and in overestimation of the aortic valve area.

**Learning Objectives**

• To understand that multiple-window interrogation is usually required but not systematically implemented in routine practice. Moreover, its role has been rarely described for contexts other than the Doppler evaluation of aortic valve stenosis.
• To identify settings in which the often-forgotten non-apical echocardiographic approach can help in the diagnosis.
• To improve the diagnostic accuracy of echocardiography for the evaluation of the aortic valve, left ventricular outflow tract flow acceleration, and the ascending-aorta by routinely implementing the non-apical windows interrogation.

Hatle et al^1^ established the utility of routine Doppler interrogation from multiple acoustic windows (suprasternal notch, right and left sternal-border, apex, and subxiphoid) to detect the maximal aortic flow velocity in aortic stenosis (AS). Accordingly, Stamm et al^2^ reported the suprasternal/supraclavicular area as the most common approach to detect the maximum aortic velocity. Subsequent studies examining the frequency with which the peak aortic velocity is obtained from the standard imaging windows found that the apical window was the most common location of the highest jet velocity.^3^^,^^4^ However, these studies involved patients younger than those in contemporary AS cohorts.

In elderly patients, acute angulation of the aortic root has been described.^5^^,^^6^ This altered spatial orientation of the aortic root could generate a more anteriorly directed stenotic jet in AS, making it difficult to sample from the apical position.

Disappointingly, the multiple-imaging window approach is not widely implemented in contemporary routine practice, favoring the convenient shortcut of the sole apical view.^7^ This practice may lead to systematic underestimation of the transaortic jet velocity, enhancing the disagreement among different parameters of AS severity.^8^, ^9^, ^10^

There are other clinical contexts aside from AS in which multiwindow interrogation might help to perform a more refined evaluation, including left ventricular outflow tract (LVOT) flow acceleration and the ascending aorta.

This case series aims to emphasize the pivotal importance of multiwindow interrogation for the assessment of the aortic valve, LVOT level, and the aorta. Written consent and ethical oversight were obtained.

## Case 1: tailoring the follow-up for moderate AS

**Figure 1:**
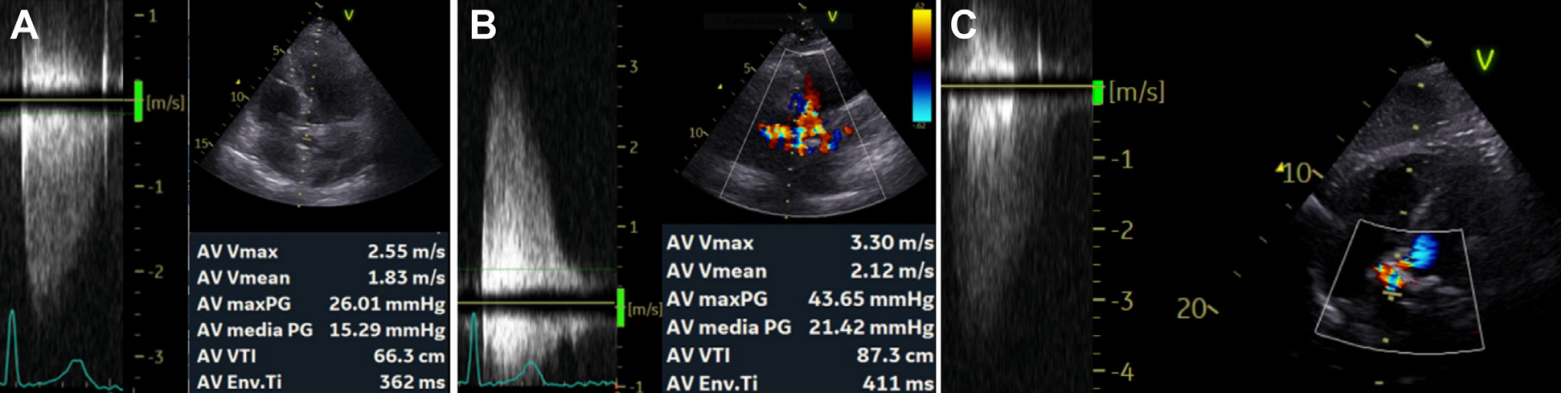
Moderate Aortic Valve Stenosis The right parasternal approach (B) and sometimes the subcostal view (C) can help to avoid the misclassification of aortic stenosis that is otherwise graded as mild from the apical view (A). AV Env.Ti = aortic envelope-time; AV maxPG = aortic peak gradient; AV media PG = aortic mean gradient; AV Vmax = aortic maximum velocity; AV Vmean = aortic mean velocity; AV VTI = aortic velocity-time integral.

A 68-year-old asymptomatic female without cardiovascular risk factors and a previous diagnosis of mild AS during a transthoracic echocardiography performed 4 years before was studied for her scheduled follow-up from the apical 5-chamber view; velocities and gradients were consistent with mild AS (Figure 1A). Conversely, Doppler measurements obtained from the right parasternal approach (RPA) revealed moderate AS (Figure 1B) which required closer-follow-up.

In other patients, the best alignment can be obtained by the subcostal approach (as shown for a different case in Figure 1C).

## Case 2: management implications of severe aortic stenosis

**Figure 2:**
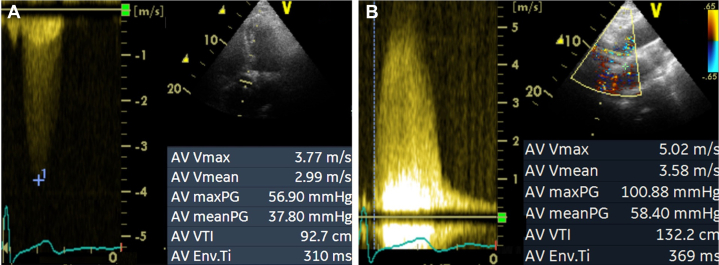
Severe Aortic Valve Stenosis The right parasternal approach identified a very severe stenosis (B) vs the apical view (A).

A 76-year-old asymptomatic male was referred to the laboratory by the general practitioner after first evidence of a 4/6 high-pitched systolic murmur. Figures 2A and 2B show the continuous-wave Doppler tracings obtained from the apical 5-chamber view and RPA, respectively. By sampling the jet velocity from the RPA, the diagnosis shifted from moderate to very-severe AS with immediate clinical consequences.

An evaluation for aortic valve replacement (AVR) should be considered in asymptomatic patients with very severe AS (ie, transaortic peak velocity >5 m/s or mean transaortic gradient ≥60 mm Hg) with low procedural risk.

## Case 3: avoid misclassification of low-gradient AS

**Figure 3:**
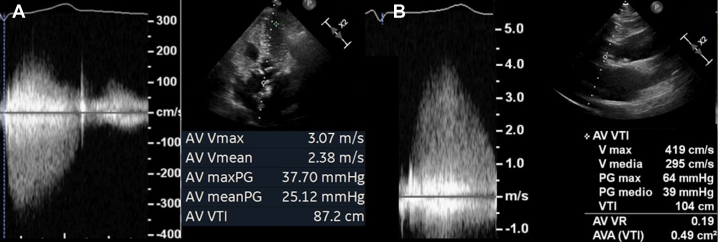
Apparent Low-Flow Low-Gradient Aortic Stenosis The right parasternal approach (B) solved the aortic valve area (AVA)/gradient inconsistencies (A).

A 78-year-old female with exertional dyspnea, ankle edema, and a mesocardial 3/6 systolic murmur underwent echocardiography. Left ventricular ejection fraction was 55%. From the apical 5-chamber view, the peak transaortic velocity was found to be 3.1 m/s, the mean gradient 25 mm Hg, the aortic valve area (AVA) 0.8 cm^2^, and stroke volume index 32 mL/m^2^. Values were consistent with low-flow low-gradient AS (Figure 3A). However, Doppler sampling from the RPA recorded a peak velocity of 4.1 m/s, mean gradient of 39 mm Hg, AVA of 0.5 cm^2^, resolving the AVA/gradient discordance and confirming AS severity (Figure 3B).

In this setting, the RPA prevented the need for further diagnostic exams (ie, computed tomography for calcium score or stress echocardiography) to clarify the AS severity grade.^11^

## Case 4: explaining symptoms of aortic bioprosthesis degeneration

**Figure 4:**
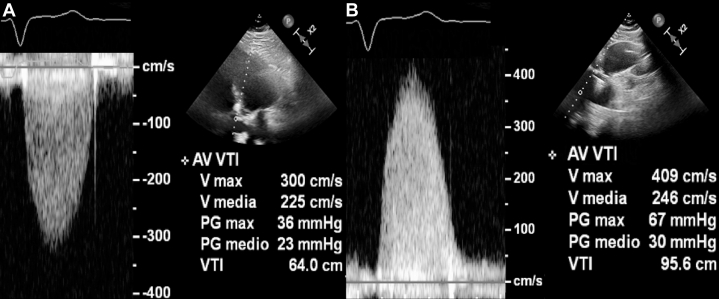
Aortic Bioprosthesis Degeneration The right parasternal approach (B) revealed higher gradients through the bioprosthesis vs the apical view (A).

A 56-year-old male underwent a prior AVR procedure (PERIMOUNT Magna 27 mm), for the stenotic bicuspid aortic valve. At follow-up, the patient had worsening dyspnea (NYHA functional class III) and a 3/6 systolic murmur. Echocardiography revealed abnormal hemodynamic parameters of prosthesis from the apical 5-chamber view (Figure 4A). However, Doppler evaluation from the RPA identified even higher velocities and gradients through the bioprosthesis that was consistent with prosthesis degeneration (Figure 4B). Despite a slightly reduced feasibility of the RPA after AVR, Doppler evaluation can improve the hemodynamic assessment of prosthetic valves. The RPA is the best acoustic imaging approach in patients with severe AS, and it generally remains so even after valve replacement.^12^

## Case 5: hypertrophic cardiomyopathy: LVOT obstruction

**Figure 5:**
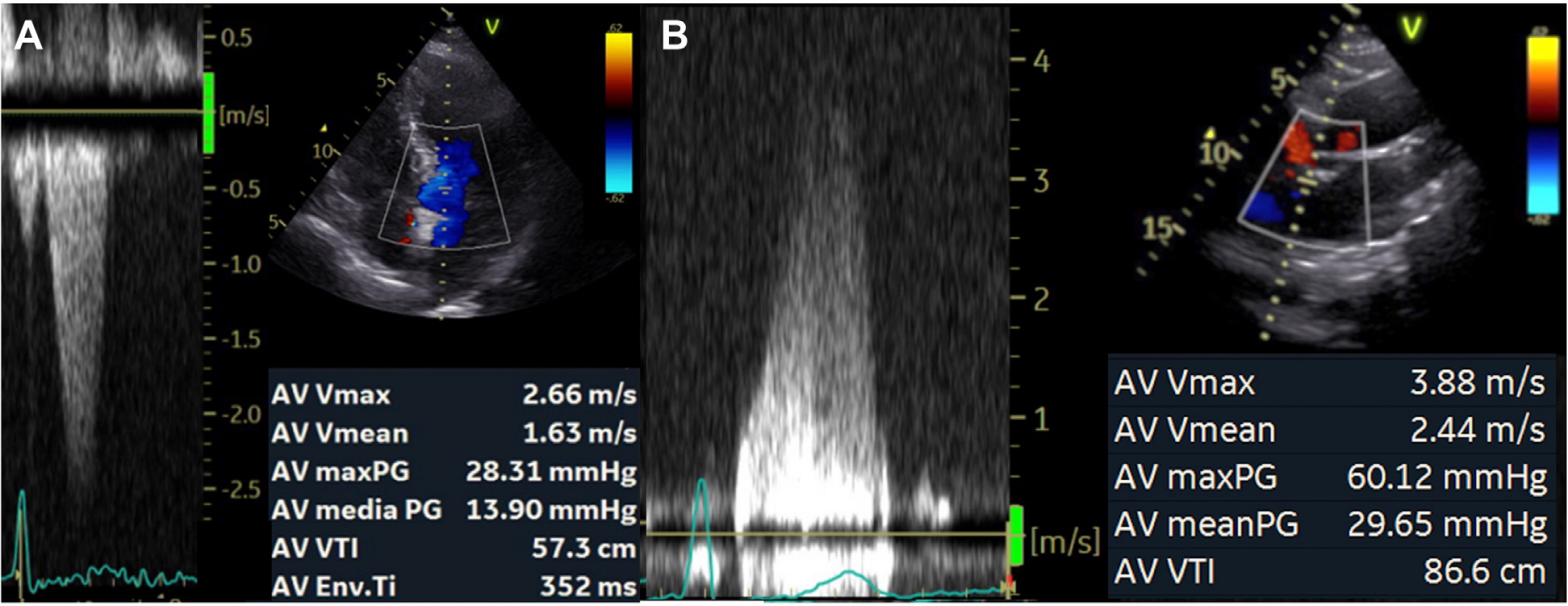
Left Ventricular Outflow Tract Obstruction Left ventricular outflow tract obstruction as assessed from the apical (A) and right parasternal (B) views.

In a 45-year-old female with signs of chest congestion, echocardiography revealed septal thickness of 18 mm and systolic anterior motion of the mitral valve which allowed determination of moderate regurgitation. Doppler assessment from the apical 5-chamber view detected a maximum gradient of 28 mm Hg and a maximum velocity of 2.7 m/s during the Valsalva maneuver (Figure 5A). However, the RPA showed more pronounced obstruction with a higher peak gradient and velocity with a similar Valsalva maneuver (60 mm Hg, 3.8 m/s) (Figure 5B). This may have profound implications in risk stratification for sudden death as well as in defining the effectiveness of medical treatment on LVOT obstruction. In this case, a beta-blocker was introduced and a strict follow-up was planned.^13^ Suboptimal evaluation could reduce the ability to refer for interventions targeting LVOT obstruction.

## Case 6: aortic dissection: detecting the flap

**Figure 6:**
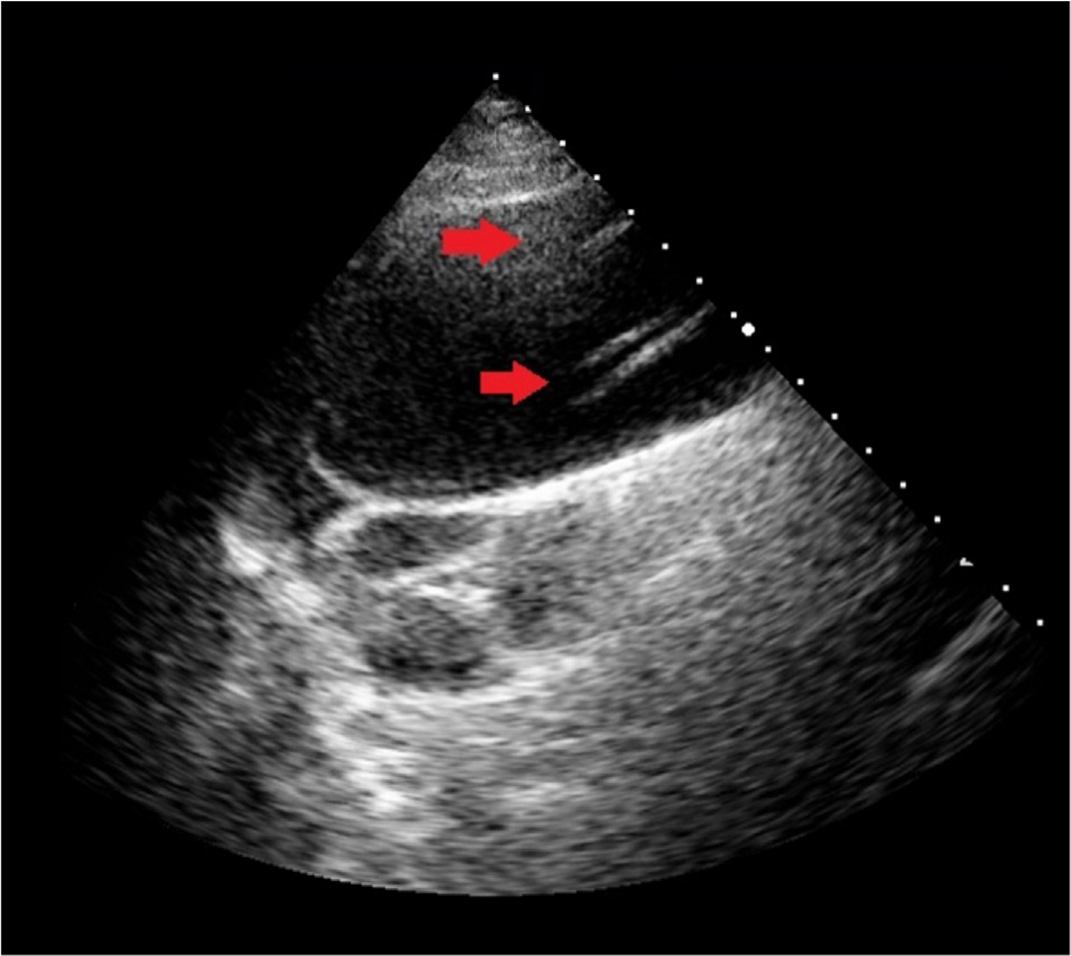
Aortic Dissection The right parasternal view definitively highlighted the intimal flap (red arrows).

A 65-year-old female was urgently admitted to the emergency room with chest pain and progressing hemodynamic instability. Point-of-care ultrasound from the RPA showed evidence of aortic dissection with an intimal flap. The left parasternal approach demonstrated limited feasibility in detecting the intimal flap in this case, necessitating a non-conventional echocardiographic approach (Figure 6). RPA allows assessment from a perpendicular perspective compared to the parasternal long-axis view and could corroborate the differential diagnosis of acute chest pain.

## Case 7: dilatation of the aortic root and ascending-aorta is important in repeated exams

**Figure 7:**
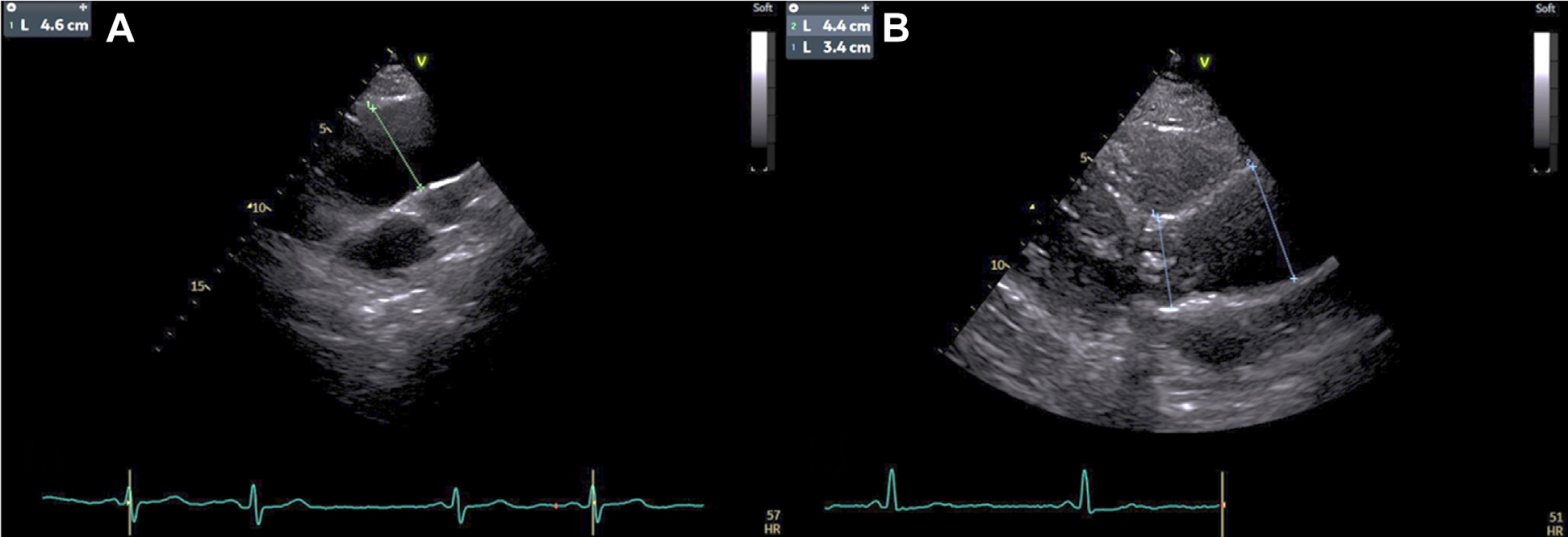
Ascending Aorta and Aortic Root Dilation The right parasternal view (B) allowed visualization of a more distal tract of the aorta vs parasternal long-axis view (A).

A 67-year-old hypertensive asymptomatic male underwent cardiologic screening. Echocardiography from the RPA revealed a dilatated ascending aorta that was more precisely visualized in this perspective compared to the parasternal long-axis view (Figures 7A and 7B). RPA might be valuable for the screening of the extent of the aortic dilatation, particularly in asymmetric cases, before requesting an eventual computed tomography scan.

## Case 8: assess the flow for the left ventricular assist device

**Figure 8:**
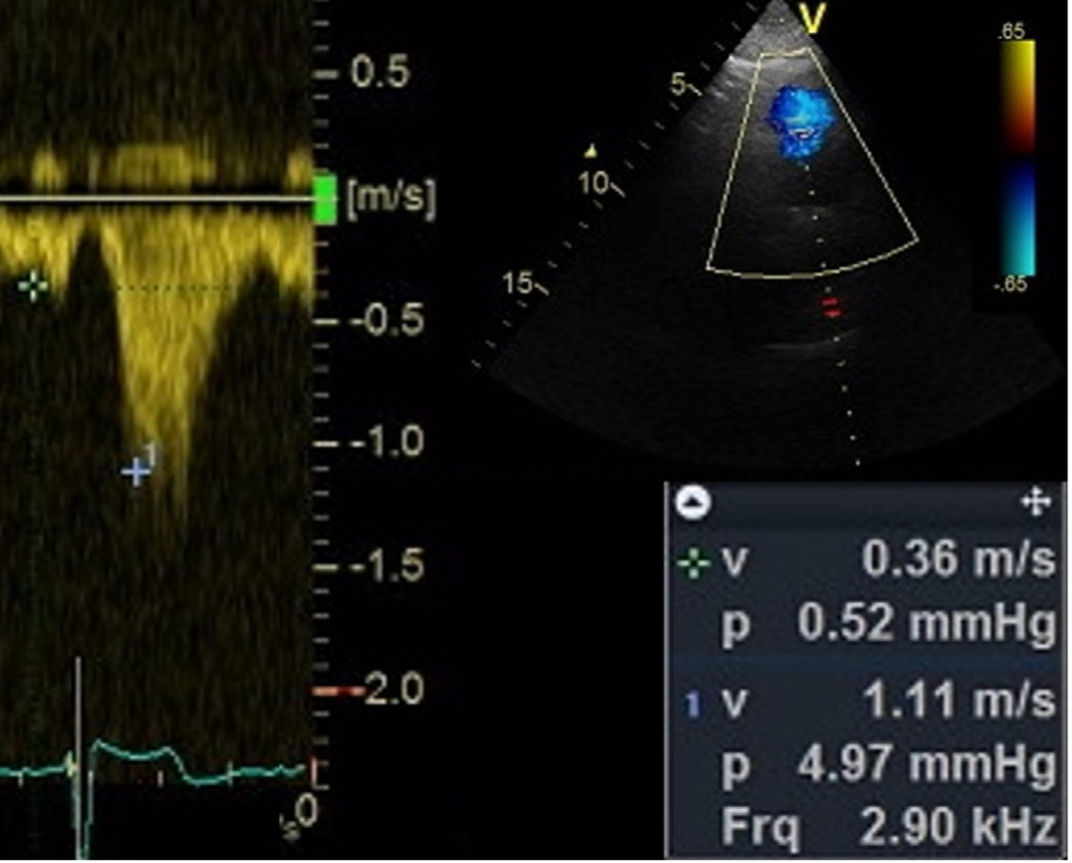
Left Ventricular Assist Device The systolic-diastolic velocity measurements are sampled in the outflow canula by pulsed-wave Doppler imaging from the right parasternal view.

A 72-year-old male with end-stage heart failure secondary to ischemic cardiomyopathy underwent implantation of a left ventricular assist device (LVAD) (HeartMate, Abbott). Post-procedural echocardiographic examinations were performed to guarantee the pump optimal settings and exclude adverse events. One key step is the interrogation of the outflow graft in the ascending aorta which usually requires RPA with color Doppler and spectral Doppler interrogations (Figure 8).^14^

## Funding Support and Author Disclosures

The authors have reported that they have no relationships relevant to the contents of this paper to disclose.
